# Arresting metastasis within the microcirculation

**DOI:** 10.1007/s10585-021-10109-8

**Published:** 2021-07-09

**Authors:** Angelos Varotsos Vrynas, Julia Perea Paizal, Chris Bakal, Sam H. Au

**Affiliations:** 1 Department of Bioengineering, Imperial College London, London, SW7 2AZ UK; 2 Institute of Cancer Research, 237 Fulham Road, London, SW3 6JB UK

**Keywords:** Circulating tumour cells, Metastasis, Cancer, Biomechanics, Microvasculature, Immune cells, Platelets

## Abstract

The behaviour of circulating tumour cells in the microcirculation remains poorly understood. Growing evidence suggests that biomechanical adaptations and interactions with blood components, i.e. immune cells and platelets within capillary beds, may add more complexity to CTCs journey towards metastasis. Revisiting how these mediators impact the ability of circulating tumour cells to survive and metastasise, will be vital to understand the role of microcirculation and advance our knowledge on metastasis.

## From microcirculation to metastasis

**Fig. 1:**
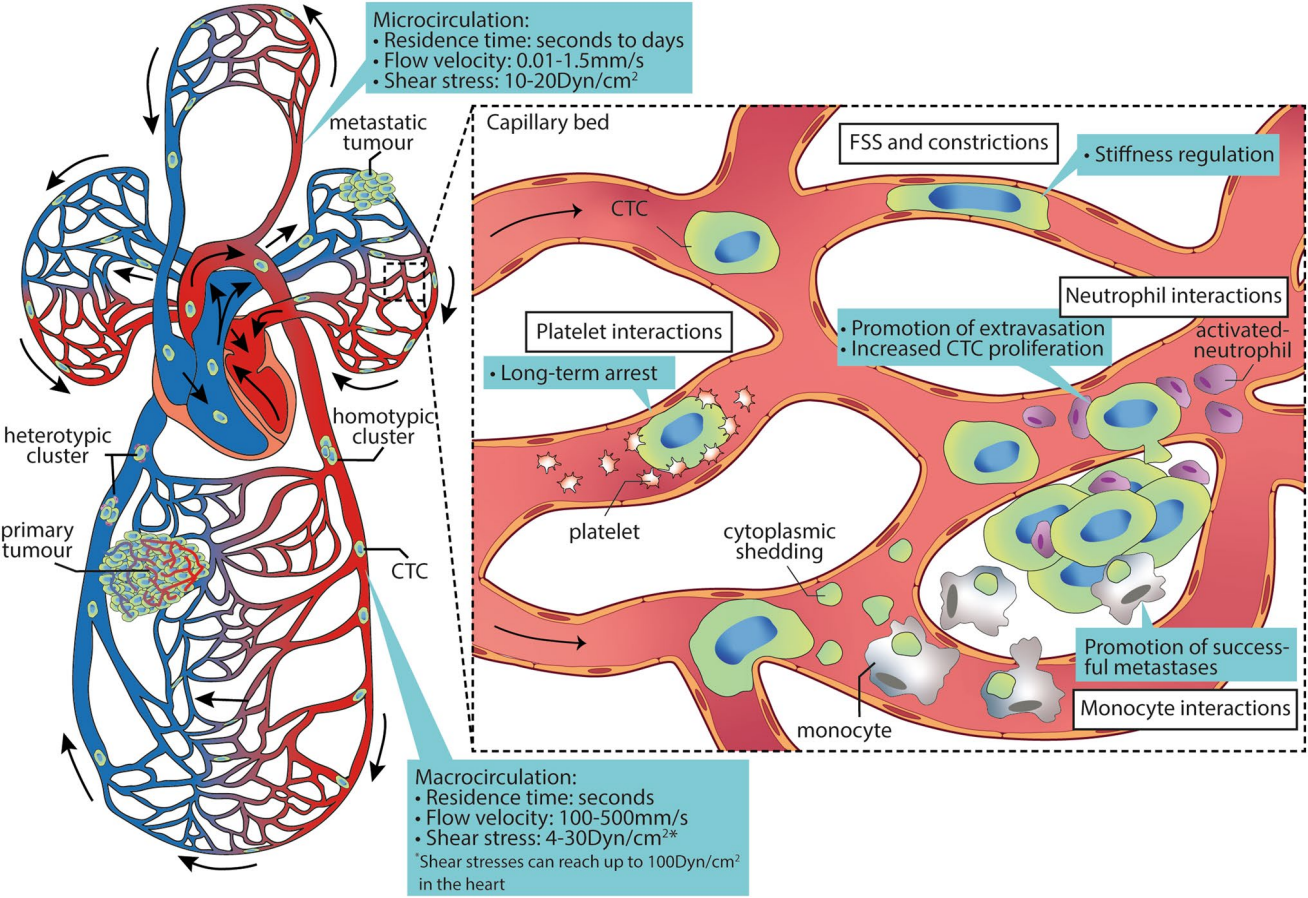
Tumour cell adaptations and interactions within the microcirculation. Cancer cells escape from primary tumours and are shed into the circulation, where they eventually arrest in capillary beds. Inset: Transit through the microcirculation alters CTC behaviours. Cell deformation due to biomechanical forces (FSS, constrictions) may induce phenotypic changes on CTCs, such as adaptations in cellular stiffness. Moreover, interactions of CTCs with immune cells, endothelium and platelets also take place, leading to stable arrest (platelets, neutrophils), extravasation (neutrophils) and colonization (monocytes and neutrophils)

The metastatic process is staggeringly inefficient. Estimates in animal models suggest that a single gram of primary tumour tissue can shed more than four million cells in a single day [1], yet few metastatic tumours are seeded by these cells. Recent evidence suggests that the ability of cancer cells to metastasize is significantly influenced by events that occur in the microcirculation. The network of small vessels and capillaries that comprise the microcirculation present a unique blood microenvironment with extensive branching and narrow constrictions capable of slowing and trapping CTCs [2]. Previous studies have shown that passive size restriction was a major determinant in the lodgement of CTCs within the microcirculation [3, 4], yet more recent studies suggest otherwise [2]. The residence time of CTCs within the microcirculation varies from hours up to days[5], allowing for sufficient exposure time of CTCs to several microenvironmental cues (Fig. 1). In this commentary, we cover recent studies that describe the pro-metastatic role of microcirculation on metastasis, including the induction of biomechanical adaptations and blood cell-CTC interactions during their transit, warranting future research to address these underrepresented concepts in the context of metastasis.

## Biomechanical forces shape CTC during capillary transit

Capillaries, the narrowest blood vessels in the human body, are smaller than the diameter of the average tumour cell. Mechanical deformation, together with fluid shear stresses (FSS) induced by the frictional forces of fluids passing tangentially over the surface of CTCs, can damage or even destroy tumour cells during capillary transit [6]. However, recent evidence suggests that these forces may as well increase the metastatic potency of cancer cells.

Two recent studies [7, 8] have suggested that tumour cells may adapt to FSS and biomechanical constriction forces by activation of the RhoA-ROCK pathway, a key regulator of cell cytoskeleton in eukaryotic cells involving the signalling protein GTPase RhoA. Once activated, RhoA recruits and activates downstream effectors proteins, such as the Rho-associated protein kinase (ROCK), which in turn, upregulates cell contractility by contracting actomyosin networks. In one study, tumour cells acquired the ability to withstand subsequent FSS damage by RhoA activation [8]. Upon activation, RhoA modulated cytoskeletal contractility through F-actin and actomyosin, enhancing the intrinsic ability of tumour cells to survive haemodynamic forces, favouring metastasis. In support of this assertion, tumour cells injected into mice models and treated with blebbistatin, an inhibitor of the RhoA downstream effector myosin II, exhibited reduced intravascular survival and subsequently arrest, which could explain the delay on metastatic onset that was observed. RhoA has also been shown to get activated by mechanical stress in the microvasculature [7]. When tumour cells transited zebrafish and mouse microvasculature, RhoA-ROCK dependent actin filament polymerization induced a change of the CTCs shape from elongated to spherical, promoting a stable arrest, cell survival and enhanced extravasation of CTCs. These conferred advantages by RhoA likely explain the increase in incidence of metastatic lesions, though it did not affect tumour cell proliferation within them. Overall, RhoA seems to be an important pathway exploited by CTCs to overcome metastatic inefficiencies imposed by FSS and lack of stable arrest.

Another mediator of cellular motility and proliferation is the Yes-associated protein 1 (YAP1) and its paralog TAZ, downstream effectors of the Hippo pathway. Although previously reported as metastatic drivers, their effects on CTCs behaviour within the microcirculation remain elusive. Interestingly, Benjamin et al. [9] have recently reported how YAP activation affects the dissemination patterns of metastasis. Using zebrafish and mice models, Benjamin et al. observed that mutated cancer cells in which YAP was insensitive to the Hippo pathway inhibition, promoted metastasis in distant organs such as the brain [9]. Yap-mutated cells showed an enhanced capability of escaping the first encountered capillary bed and re-entering the systemic circulation. This resulted in their arrest in the brain microvasculature, in opposition to control cells, which displayed lasting arrest in the first capillary bed. Although the authors did not evaluate the behaviour of YAP-inhibited cells, they observed that intravenous injected cells with the mutated YAP pathway resulted in a fivefold increase of the brain tumour burden in mice when compared to wild-type ones. This suggests that YAP might be activated by the characteristic microenvironment of microcirculation through FSS, nuclear deformation and the interaction with platelets [9]. However, the authors did not specify how cells with a more contractile phenotype, expected upon YAP activation, could escape capillary beds. Paradoxically, another recent study [10] reported a decrease in cell stiffness on cells traversing capillaries, induced by YAP deactivation. Although this study did not include fluid flow, the confined channels had a comparable size to these encountered in the microcirculation (5 μm) and cell stiffness regulation was tracked in situ through AFM, suggesting that cancer cells may dynamically regulate their stiffness when transiting capillaries. However, this study contradicts Huang et al*.*, which argues that the constricted environment of capillaries had the opposite effect, i.e. an increase of cell contractility.

Another factor that needs to be considered on CTCs ability to migrate through constricted vessels is cell heterogeneity. Using microfluidic models of capillary-sized constrictions, Cognart et al*.* demonstrated that FSS and constriction forces induced different biomechanical effects on tumour cells of different phenotypes [11]. The mesenchymal cell line they used exhibited increased deformability during microchannel transit when compared to the epithelial line. These results propose that cells might modulate a different response when transiting capillaries depending on their phenotype and that this heterogeneity might eventually affect their metastatic potential. While in this study different cell lines from different subgroups were compared (i.e. triple negative mesenchymal-like vs. HER2 + epithelial-like cells), what made the comparison challenging, there is also evidence that CTCs from the same patient experience changes in their EMT phenotype as they transit through the microcirculation. In fact, Sun et al. showed a predominant epithelial phenotype in patient-derived CTCs after their release from the primary tumour, while a mesenchymal phenotype was more abundant on CTCs collected after crossing capillary beds [12]. In summary, the stiffening of CTCs within the microcirculation may be an important step in metastatic success, yet it is not well-established in the context of mechanical adaptation (e.g. by modulation of RhoA, YAP), CTC heterogeneity and EMT. CTCs may find it favourable to switch between softer and stiffer phenotypes at different times within the circulation. For instance, they have to be able to resist compression and fluid stress by regulating different signalling pathways such as Rhoa and YAP, but also be able to acquire softer phenotypes that may promote capillary migration, extravasation and invasion. Thus, since there is no current consensus on how this switch is regulated, future research should focus on answering how CTCs dynamically regulate their stiffness on the different steps of the metastatic cascade, what may provide us key insights into the drivers of CTC invasion and survival.

## The crosstalk between immune cells and CTCs: a contribution to metastasis

While much research has been conducted exploring the role of immune cells in the progression of primary tumours, the interactions of immune cells with tumour cells within the microcirculation are largely unexplored. As demonstrated by Headley et al., CTCs that arrested in bifurcated lung microvessels in mice experienced FSS-dependent generation of cytoplasmic fragments [13]. The size of these fragments varied from 0.5 to 25 microns in diameter and were frequently ingested by myeloid cells including neutrophils and monocytes. While the authors did not examine the impact of this ingestion process on the phenotypes of immune cells, they did observe an increased trend of extravasation by monocytes and macrophages that ingested tumour fragments. However, the molecular link between this immune behaviour and the establishment of metastases remains elusive.

Neutrophils, the most abundant circulating leukocyte, have also been implicated in CTC interactions within the microcirculation. A recent study by Chen et al*.* using a self-organised perfusable microvasculature identified enhanced heterotypic aggregation upon injection of tumour cells and inflamed neutrophils [14]. Local release of chemotactic factors by tumour cells and neutrophils from these aggregates recruited additional neutrophils, which promoted endothelial retention of CTCs in the microvasculature. The authors suggested that the recruited neutrophils increased flow resistances by occluding the area around the aggregates, possibly preventing re-entry of tumour cells in systemic circulation. Coupled with the fact that neutrophils secreted cytokines that disrupted endothelium integrity, these indications may explain why more extravasation events were observed by tumour cells when they were co-injected with inflamed neutrophils. Interestingly, in another study [15] neutrophils enhanced tumour cell arrest in the microvasculature of mice models and further protected CTCs from immune attack by inhibiting the activation of a natural killer cell-dependent immune response, reducing thus their clearance. Moreover, neutrophils played a role in disrupting endothelial barriers by secreting Interleukin 1-β (Il-1β), promoting thus extravasation of tumour cells. Overall, these studies highlight how immune interactions may increase the availability of tumour cells in the microcirculation, leading to increased metastases development.

While promoting extravasation may increase the likelihood of metastasis, the proliferative abilities of CTCs post-extravasation is a key step in metastasis. Szczerba et al. recently identified a crucial role of neutrophils in this process by demonstrating that heterotypic neutrophil-tumour cell clusters had greater metastatic aggressiveness than single or homotypic clusters [16]. During dissemination, tumour cells that were associated with neutrophils retained higher proliferative ability compared to neutrophil-free CTCs. Interestingly, they found out that this increase was only evident in circulation, despite neutrophil proximity on tumour cells in primary or secondary organs. To further explore how neutrophils enhance the metastatic ability of tumour cells, the authors pre-treated for 24 h tumour cells with cytokines such as Il1-β or Interleukin 6 (Il-6), which are often secreted by neutrophils, and found out that inoculation of cytokine-treated tumour cells on animals led to more aggressive metastases and faster demise. This experimental evidence does not differentiate whether this change in CTCs proliferative ability is reflected from the neutrophil-tumour cell interactions within the primary tumour or adopted in the short half-time of clusters in circulation. However, it is noteworthy to mention that clusters may be entrapped for hours in the microcirculation, providing sufficient time for neutrophils to preserve or promote the proliferative ability of tumour cells and may explain the sharp increase in proliferation observed away from the primary tumour. Nonetheless, these interactions may be further complicated by another blood component, i.e. platelets.

## Platelets: an intercellular ‘bridge’ in the microcirculation

Circulating tumour cells are often found coated by platelets. Their versatile role in conferring protection to CTCs and facilitating metastasis includes inhibition of cytotoxic immune recognition, recruitment of pro-metastatic leukocytes, endothelium arrest, extravasation, EMT and angiogenesis, as reviewed by Gkolfinopoulos et al. [17]. Particularly, intravascular aggregation of CTCs and platelets has been recorded in recent studies in vivo, which highlight the crucial role of heterotypic CTC-platelet aggregates in microcirculation arrest [18–20], yet its implication to metastasis remains less understood.

To understand the impact of platelets on metastasis, Echtler et al*.* examined initially the role of platelets on tumour cell arrest [18]. A key molecular driver of platelet aggregation process appears to be glycoprotein IIb (GP IIb). Using flow chamber assays that mimicked capillary shear rates, they observed that the presence of platelets, positive or negative for GP IIb, did not affect tumour cell attachment to endothelial cells. Instead, GP IIb + rather than GP IIb- platelets promoted both in vitro and in vivo the formation of heterotypic clusters of melanoma cells and platelets. These larger heterotypic aggregates, as the authors suggested, played a role in physical occlusion through size restrictions at the microvasculature, favouring their arrest. The importance of platelet-CTC aggregates in metastasis is further supported by evidence in mice that were treated with antiplatelet agents, such as ticagrelor [19] and aspirin [20], which led to a decreased metastatic burden. Both studies suggest that this inhibitory impact on metastasis was due to a reduction in the intravascular aggregation between platelets and CTCs that affected the ability of CTCs to arrest or remain trapped. Particularly, Lucotti et al*.* attributed this aggregation to activation of platelets through cyclooxygenase (COX-1) signalling pathway and further linked this to downstream factors that may correlate to successful metastasis, such as endothelium arrest, endothelium activation and monocyte recruitment. Interestingly, the conflicting results in the platelets role on CTCs attachment to the endothelium presented by Echtler et al. [18] and Lucotti et al. [20] may be explained by the fact that each study employed a different mechanism driving platelet activation (i.e. GP IIb vs COX-1 respectively).

Thus, antiplatelet agents targeting different platelet activation pathways or steps of the metastatic cascade *i.e*. arrest, may help identify the distinct contributions deployed by platelets to drive metastasis. Moreover, platelets are also involved in the activation of biomechanical pathways relevant to tumour cells such as RhoA or Yap [21], whose impact on metastasis was described in previous sections. This suggests that platelets may be more versatile than initially believed, influencing not only intercellular interactions but also biomechanical adaptations. In summary, platelets may “bridge” the interactions between different tumour cell phenotypes, immune cells and the endothelium within the unique blood microenvironment of capillary beds.

## Discussion

Although the metastatic journey of CTCs is highly inefficient, development of metastases is still inevitable in many cancer patients. Our inability to understand which factors select a minority of cancer cells to metastasise is partially attributed to the unexplored dynamic contributions of biomechanics, intercellular interactions and biochemical signalling within physiologically relevant models. Advances in CTC and CTC cluster isolation from the blood of cancer patients [16, 22, 23], together with the improvement of models with human-mimicking blood flow velocities, microvessel architecture and representative immune populations will be vital to answer more complex questions of CTCs behaviour.

First, how do FSS and confinement modulate CTC stiffness at the different steps of the metastatic cascade? Following this, how do stiffness dynamics affect CTC arrest, extravasation and metastatic potential? Second, how will CTC heterogeneity (e.g. a more epithelial vs a more mesenchymal phenotype) affect their ability to migrate through the confined environment of capillaries? Third, how do the various immune and/or platelets-CTC interactions in the microcirculation promote or inhibit metastasis across different cancers? Last but not least, how will the individual adaptations acquired by CTCs in the microcirculation, such as phenotypic changes, affect cell fate after extravasation. Understanding how these individual factors enable CTCs to overcome metastatic inefficiency will bring us a step closer to comprehend how they may collectively affect the metastatic probabilities. This will ultimately allow us to predict which cancer cells will succeed on their metastatic journey, and consequently, improve our chances to arrest metastasis.
